# Pilot Study on the Prevalence of Diamine Oxidase Gene Variants in Patients with Symptoms of Histamine Intolerance

**DOI:** 10.3390/nu16081142

**Published:** 2024-04-12

**Authors:** Adriana Duelo, Oriol Comas-Basté, Sònia Sánchez-Pérez, M. Teresa Veciana-Nogués, Eva Ruiz-Casares, M. Carmen Vidal-Carou, M. Luz Latorre-Moratalla

**Affiliations:** 1 Departament de Nutrició, Ciències de l’Alimentació i Gastronomia, Campus de l’Alimentació de Torribera, Universitat de Barcelona (UB), Av. Prat de la Riba 171, 08921 Santa Coloma de Gramenet, Spain; aduelo@ub.edu (A.D.); soniasanchezperez@ub.edu (S.S.-P.); veciana@ub.edu (M.T.V.-N.); mcvidal@ub.edu (M.C.V.-C.); mariluzlatorre@ub.edu (M.L.L.-M.); 2 Institut de Recerca en Nutrició i Seguretat Alimentària (INSA·UB), Universitat de Barcelona (UB), Av. Prat de la Riba 171, 08921 Santa Coloma de Gramenet, Spain; 3 International Institute of DAO Deficiency, C/Escoles Pies 49, 08017 Barcelona, Spain; 4 Vivolabs, C/Marqués de la Valdavia 106, 28100 Alcobendas, Spain; eva.ruiz@grupo-vivo.com

**Keywords:** histamine, diamine oxidase (DAO), *AOC1* gene, histamine intolerance

## Abstract

A retrospective pilot study was carried out to investigate the prevalence of four variants of the diamine oxidase (DAO) encoding gene (*AOC1*) in Caucasian adults with symptoms of histamine intolerance. In a cohort of 100 patients and 100 healthy individuals, DAO-encoding gene non-synonymous Single Nucleotide Variations (SNVs) were genotyped by multiplex single-nucleotide primer extension (SNPE) and capillary electrophoresis, and serum DAO activity was analyzed with a radio-extraction assay. The study found that 79% of individuals with symptoms of histamine intolerance harbored one or more of the four SNVs associated with reduced DAO activity. No significant differences were found in the prevalence of any variant between the group of patients and healthy controls. However, when considering the status of the alleles associated with DAO deficiency, more homozygous alleles were observed in histamine-intolerant patients. Moreover, a slightly but statistically higher percentage of patients had a high genetic risk score, reflecting the cumulative effect of carrying multiple DAO deficiency-associated gene variants and a high load of risk alleles (homozygous). A relationship between serum DAO activity and the genetic load of one specific SNV was observed, with DAO activity being significantly lower in patients homozygous for rs2052129. These results potentially support that carrying multiple DAO deficiency-associated gene variants and a high load of risk alleles (homozygous) is more relevant than the mere presence of one or more SNVs. Further studies are needed to determine the predictive value of these DAO-encoding gene variants.

## 1. Introduction

Histamine is a biogenic amine involved in many essential physiological functions due to its interaction with four G-protein-coupled receptors with seven transmembrane domains (H1, H2, H3 and H4), which activate signal transduction pathways upon perceiving their ligand, histamine [1,2,3]. This amine plays a crucial role in various immune and physiological mechanisms, stimulating gastric acid secretion, inflammation, smooth muscle cell contraction, vasodilation and cytokine production, among other processes [2,3]. Moreover, histamine acts as a neurotransmitter synthesized by neurons located in the posterior region of the hypothalamus, whose axons extend through the brain [3].

Histamine is also found in a wide range of foods due to the action of several fermentative or spoilage bacteria [3]. The effect of dietary histamine on individuals with a low ability to metabolize it, a condition known as histamine intolerance, has been investigated in the last two decades [3]. A deficiency in the catalytic activity of the enzyme diamine oxidase (DAO), a secretory protein responsible for the gastrointestinal degradation of exogenous histamine, seems to be the main cause of this food intolerance [2,4]. The clinical manifestations of the disorder are diverse and include a plethora of unspecific digestive, neurological, dermatological, respiratory, and cardiovascular complaints. Recent studies have shown that patients with histamine intolerance often suffer a complex combination of symptoms, the most frequently reported being abdominal distention, diarrhea, constipation, headache, migraine, flushing, pruritus, low blood pressure and rhinorrhea [3,4,5]. DAO deficiency can be acquired; for example, it may be caused by the impaired mucosal integrity associated with several inflammatory bowel pathologies or the DAO inhibitory effect of certain pharmacological drugs [6,7]. In addition, two recent studies have postulated that dysbiosis of the intestinal microbiota may also trigger symptoms of histamine intolerance, although more research is needed to determine whether the alteration of the gut microbiota is a cause or consequence of this food intolerance [8,9]. 

DAO deficiency may also have a genetic origin [10,11,12]. To date, more than 50 non-synonymous Single Nucleotide Variation (SNVs) have been identified in the coding sequence of the DAO-encoding gene (*AOC1/ABP1*) located in chromosome 7 (7q34-q36) [11,12,13,14,15]. Among them, rs10156191, rs1049742 and rs1049793 are reported to be the SNVs that most affect DAO functionality in Caucasian individuals [6,12,15,16]. Moreover, a SNV in the promoter region of the DAO-encoding gene (rs2052129) is associated with reduced transcriptional activity [16]. Some other genetic variations related to histamine metabolism have been described in Asian (rs45558339) and African (rs35070995) populations [11]. 

Several recent works have investigated the correlation between reduced DAO activity and certain genetic factors. Ayuso et al. (2007) observed that carriers of the variant rs1049793, whether heterozygous or homozygous, displayed a lower serum DAO activity than non-carriers [6]. Similarly, Maintz et al. (2011), found that reduced DAO activity was associated with certain AOC1 variants, especially in those individuals who were carrying the T allele in variant rs2052129 [15]. However, limited existing literature delves into the examination of the association between these SNVs and the clinical manifestations of histamine intolerance. Some noteworthy examples include the research conducted by García-Martin et al. (2015), which identified a connection between variants rs10156191 and rs2052129 in patients diagnosed with migraine [16]. Likewise, another study from North India carried out by Kaur et al. (2020) reported the same connection between rs10156191 and rs2052129 variants with migraine [14]. On the contrary, the work published by Maintz et al. (2011) did not observe any significant association between *AOC1* gene SNVs and any particular symptom of histamine intolerance [15]. 

Table 1 summarizes information on the four variants of the AOC1 gene more consistently associated with DAO deficiency according to the literature [6,12,15,16]. It is important to highlight that the absence of a genetic predisposition does not exclude the possibility of suffering symptoms of histamine intolerance due to other causes.

Diagnosis of histamine intolerance is currently based on the appearance of symptoms affecting two or more organs or body systems and their improvement or remission after the dietary exclusion of histamine-containing foods [18]. Additionally, food allergies or underlying systemic mastocytosis must be ruled out. A range of complementary tests have been proposed to obtain a marker to confirm the diagnosis. The different approaches include the determination of DAO activity in serum or an intestinal biopsy sample, the identification of certain SNVs in the DAO-encoding gene, the application of a variant of the intradermal skin allergy test (histamine 50-skin-prick test), and the determination of histamine metabolites in urine samples [3,12,18,19]. In the last decade, measuring serum DAO activity has been frequently used for the complementary routine clinical diagnosis of this enzymatic deficiency, but the evidence for its utility in the diagnosis of histamine intolerance is neither abundant nor conclusive [20,21,22,23,24,25,26]. This controversy is highlighted in different articles recently published that emphasize the need for more research to verify the diagnostic value of serum DAO activity for histamine intolerance [25,26]. Despite the lack of clarity in the evidence linking symptomatology to low serum DAO activity, various studies have demonstrated that administering the DAO enzyme in supplement form significantly improved related symptoms [27,28,29,30,31]. In this context, considering the possibility that serum DAO activity has limitations as a diagnostic parameter, identifying four SNVs linked to DAO deficiency (rs10156191, rs1049742, rs1049793 and rs2052129) is currently being proposed as a novel non-invasive approach to diagnose population genetically susceptible to histamine intolerance [6,12,15,16]. 

The aim of this work was to perform a pilot study in order to assess the prevalence of the four gene variants more frequently associated with reduced DAO functionality (rs10156191, rs1049742, rs1049793 and rs2052129) in Caucasian individuals with symptoms of histamine intolerance, as well as in a group of healthy individuals. The potential correlation of the different SNVs with clinical manifestations was also evaluated, alongside the association of SNVs with DAO serum activity in patients.

## 2. Materials and Methods

### 2.1. Participants and Study Design

An observational retrospective study was carried out with 100 adult patients (female: male, 87:13; mean age, 37.8 ± 13.7 years) and 100 healthy controls (female: male, 61:39; mean age, 37.2 ± 10.8 years). Patients and healthy individuals were recruited at the International Institute of DAO Deficiency (Barcelona, Spain) and the Food and Nutrition Campus of the University of Barcelona, respectively. Patients were included only if they had two or more symptoms affecting different organs/body systems compatible with histamine intolerance (Table 2), while the control group consisted of individuals without any of the symptoms associated with this disorder. The medical history of the participants was recorded by registered dietitians specialized in DAO deficiency using an exhaustive questionnaire at the baseline visit. Participants who had been diagnosed with any allergy or were pregnant, lactating and/or taking DAO-inhibitor drugs were excluded from the study. All participants were informed in detail about the aim and procedure of the study and gave their written informed consent prior to inclusion. The study was approved by the Bioethics Committee of the University of Barcelona (IRB00003099).

### 2.2. Identification of SNVs in the DAO-Encoding Gene

The gene variants assessed in this pilot study (rs2052129, rs10156191, rs1049742 and rs1049793) have been selected because they are those associated with DAO deficiency in the Caucasian population and currently used in the clinical practice for the diagnosis of histamine intolerance [6,12,15,16].

Mucosal swab samples were used for genotyping the DAO-encoding gene’s SNVs by multiplex SNPE (single-nucleotide primer extension) and capillary electrophoresis. Four regions of interest containing the SNV in the *AOC1* gene were amplified by multiplex polymerase chain reaction (PCR). Unincorporated primers and dNTPs were removed from the PCR product by enzymatic purification. The PCR product was then used in the SNPE assay according to the manufacturer’s instructions (SNaPshot, Thermo Fisher Scientific Inc., Waltham, MA, USA), using internal primers specific for each of the variants analyzed. Separation and detection of the SNPE products were performed on a 3500 Series Genetic Analyzer (Applied Biosystems, Waltham, MA, USA). The injection cocktail, consisting of 0.3 µL of GeneScan-120 LIZ size standard and 15 µL of formamide-EDTA, was mixed with 1 µL of purified SNPE product, heat-denatured, and chilled on ice. Products were injected for 8 s at 1.6 kV, then electrophoresed for 560 s at 15 kV using Performance Optimized Polymer 7 (POP-7™, Polymer, Applied Biosystems, Waltham, MA, USA) and a 50-cm length-to-detector uncoated capillary. Data were analyzed using GeneMapper^TM^ v4.0 software (Applied Biosystems, Waltham, MA, USA).

### 2.3. DAO Deficiency Genetic Risk Score 

In order to evaluate the cumulative presence of carrying multiple DAO deficiency-associated variants, a genetic risk score was calculated. Okutan et al. (2023) previously developed and utilized this genetic DAO Score to determine the genetic load of DAO variants in fibromyalgia patients [32]. This score provides the sum of all risk alleles by giving a rating of 0 for the absence of any DAO deficiency-associated allele, 1 for the presence of one DAO deficiency-associated allele (heterozygosis), and 2 for the presence of two DAO deficiency-associated alleles (homozygosis) at each of the positions analyzed in the *AOC1* gene [32]. Based on the prevalence results obtained in the current study, the rs1049742 variant has not been considered for the calculation of the DAO deficiency genetic risk score. Moreover, to facilitate the calculation of the risk score, it was assumed that the impact on DAO activity was the same for each variant and that all of them showed a codominant genotype–phenotype correlation. This approach can be considered a limitation of the study.

### 2.4. Determination of Serum DAO Activity

Serum DAO activity was analyzed with a radio-extraction assay using a commercial kit (DAO-REA (^3^H), Immundiagnostik AG, Bensheim, Germany). The method was based on determining the reaction product, using radiolabelled putrescine-dihydrochloride as a substrate. Finally, radioactivity was determined in a beta-counter, being the signal directly proportional to the activity of DAO in the sample. Values lower than 10 U/mL were presumed to indicate DAO deficiency. One U (unit) corresponds to the DAO activity that degrades 1 μmol/mL of substrate per minute [27]. 

### 2.5. Statistical Analysis 

The statistical analysis was performed with SPSS Statistics 27.0 statistical software package (IBM Corporation, Armonk, NY, USA). Pearson’s chi-square test was used to compare the frequency of all the target SNVs between groups. The comparison of the different combinations of genotypes between the patient and control groups was performed according to Fisher’s Exact Test. Differences in the DAO activity values associated with specific gene variants were analyzed by a One-Way ANOVA test’s previous logarithmic transformation of data to ensure that it fit a normal distribution. Normality was assessed using Q–Q plots and Shapiro–Wilk’s test. Moreover, a general linear model was conducted to adjust the serum DAO activity data with age and sex as a potential confounder factor. Values of *p* < 0.05 were considered statistically significant.

## 3. Results and Discussion

### 3.1. Prevalence of SNVs in the DAO-Encoding Gene

**Figure 1:**
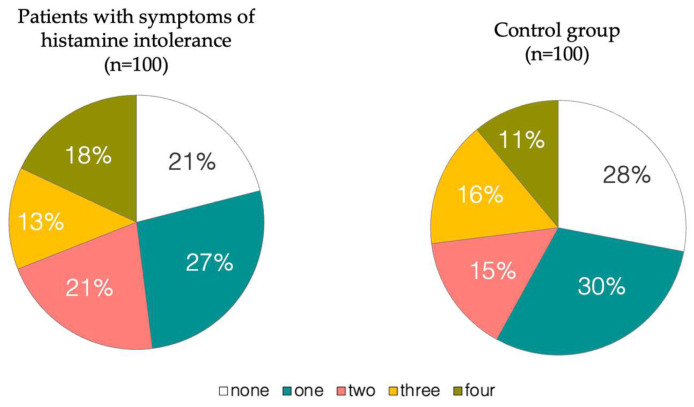
Distribution of individuals harboring different numbers of gene variants associated with reduced DAO activity.

As can be seen in Figure 1, no significant differences in the prevalence of DAO deficiency-associated SNVs (rs10156191, rs1049742, rs1049793 and rs2052129) were seen. The 79% of patients with symptoms of histamine intolerance harbored one or more of the SNVs related to reduced DAO, compared to 72% of the healthy individuals. Carriers of all four SNVs were more frequent in the patient than in the control group (18% and 11%, respectively), although without statistically significant differences (*p* = 0.16). Therefore, the simple identification of the existence of one or more allelic variants does not seem to be enough as a suitable diagnostic biomarker.

To further assess whether differences exist between groups, the prevalence of each DAO genotype and allelic variants in individuals from the two populations were analyzed (Table 3). The genotyping data were in Hardy–Weinberg’s equilibrium as the expected frequencies for wild-type, heterozygous and homozygous participants for every variant analyzed, as calculated from allele frequencies, were almost identical to those observed (*p* > 0.05). In patients with clinical manifestations of histamine intolerance, rs1049793 was found to be the most frequent variant (59%), closely followed by rs10156191 (52%) and rs2052129 (51%). In the case of rs1049742, only 18% of patients carried this allelic variant. In all cases, heterozygote genotypes prevailed. Variant frequency did not differ significantly between females and males or according to age (*p* > 0.05). The observed genotype frequencies were similar to those of previous studies [11,15,16,33,34]. Maintz et al. (2011) reported that reduced DAO activity was most frequently associated with variant rs1049793 (50.2%), closely followed by rs10156191 (43%) and rs2052129 (40%) [15], whereas the least frequent was rs1049742 (15%). A similar distribution profile of the same four SNVs was found by García-Martín et al. (2015) in patients diagnosed with migraine, a disorder often related to histamine intolerance [16], albeit with slightly lower percentages (45% for rs1049793 and rs10156191, 39% for rs2052129 and 12% for rs1049742). Very similar frequency distributions of these gene variants have been recently reported in a sample population of 296 patients diagnosed with migraine [34] and 98 women with fibromyalgia [35].

Among healthy participants, the most frequent SNV was also found to be rs1049793 (50%), followed by rs2052129 and rs10156191 (47% and 39%, respectively). As in the patients group, the rs1049742 variant in the control group was especially low (13%) (Table 3). No statistically significant differences were observed related to the sex or age of individuals (*p* > 0.05). The prevalence of all allelic variants was always lower among healthy individuals than in patients with symptoms of histamine intolerance, although no significant differences were observed between groups when comparing the individuals harboring *AOC1*-reference alleles versus *AOC1*-associated alleles (Table 3). Previous studies that included control groups have not reported any significant differences in the prevalence of any of the studied SNVs in comparison with patient groups [15,33,34,36]. 

However, when considering the state of the *AOC1*-associated alleles, more homozygous alleles were observed in the patient than in the control group. Thus, 36% of patients were homozygous for any allelic variant, compared to only 9% of healthy individuals (*p* < 0.01). Specifically, the patient group had the highest proportion of carriers of the homozygous allele at rs2052129 (19%), with none detected in the control group (*p* < 0.001). Considering that rs2052129 is located in the promoter region of the DAO-encoding gene (*AOC1*), it could have a significant impact on DAO-encoding gene expression. Homozygotes for rs10156191 were found in 12% of patients and only 4% of healthy individuals (*p* = 0.037).

Aside from the prevalence of each of the four variants (none holds statistical significance on its own), it is essential to consider the frequency of multiple variants in individuals. Among the patients carrying two gene variants, out of the six potential combinations, the most frequently found was that of rs10156191 plus rs2052129 (62%). The only combination of three genetic variants was rs10156191, rs1049793 and rs2052129. It is noteworthy that rs1049742 was only detected with the three other variants and never alone or with one or two different variants. Moreover, the prevalence of rs1049742 variant in both groups was markedly low, with a percentage below 18%. After considering these findings and earlier studies in the same vein, it can be inferred that this variant is unlikely to be associated with this disorder.

Consequently, it will not be considered from now on. Table 4 summarizes the different combinations of genotypes of three assessed variants now in both the control and patient populations. Although each variant is at Hardy–Weinberg’s equilibrium, a partial linkage disequilibrium between *AOC1* SNVs was suspected, as the frequencies of individuals carrying association of DAO-deficiency alleles were higher than those expected, as calculated from frequencies of isolated genotypes. Thus, the frequency of patients carrying the three heterozygous variants (17%) was higher than the expected frequency (<1%); and the frequency of patients carrying homozygote rs10156191 and rs2052129 and heterozygote rs1049793 was seven times the expected frequency (<1%). This last scenario is the only combination that showed statistically significant differences in comparison to the control group (*p* = 0.014).

**Figure 2:**
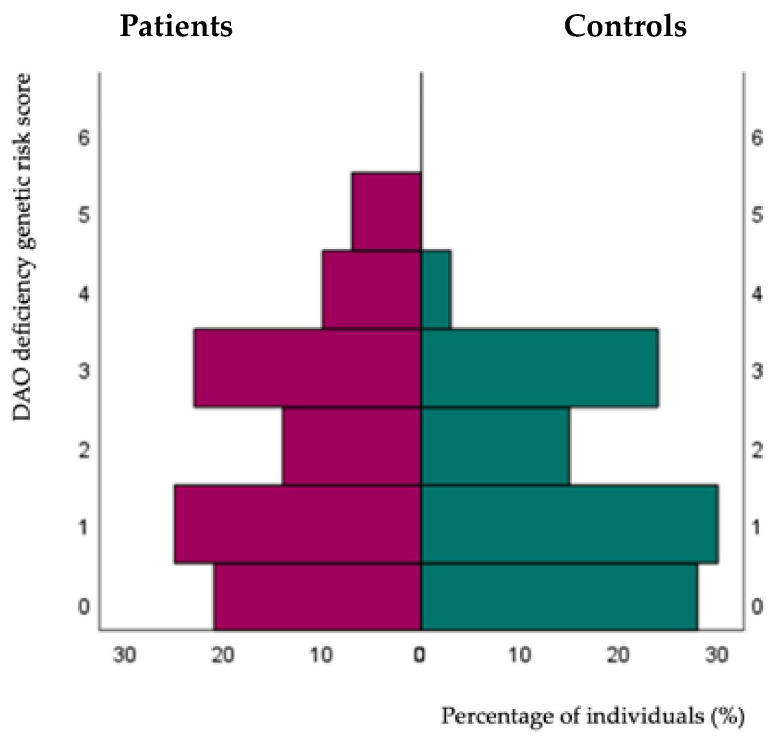
Distribution of the genetic risk scores in patients with symptoms of histamine intolerance and the control group. The distribution of values between groups was statistically different according to the Mann–Whitney U test (*p* = 0.020).

A genetic risk score was calculated to visualize the cumulative genetic load (i.e., carrying multiple DAO deficiency-associated variants plus the genotype types) according to the proposed score by Okutan et al. (2023), who applied it in patients with fibromyalgia symptoms [32]. Figure 2 compares the scores of patients with symptoms of histamine intolerance and healthy subjects. The low genetic load of the control group is striking, with low scores (0–1) achieved by 58% of individuals and none above 4. In contrast, even though the patients obtained a wide range of scores, a higher percentage had high scores (4–5) in comparison to the control group. In fact, 17% of the patients had a high genetic load in contrast to only 3% of the healthy control group. With this dataset, the optimal cut-off of balance between sensitivity and specificity was calculated, yielding a threshold value equal to or greater than 2 (sensitivity = 0.54, specificity= 0.57 and Youden Index = 0.11) to discriminate patients with symptoms related to a DAO deficiency from healthy ones. However, choosing a cut-off 4 would be more accurate, albeit lowering the sensitivity (sensitivity = 0.28, specificity = 0.88 and Youden Index = 0.16). Therefore, it could be proposed that a DAO Score equal to or above 4 would provide a more reliable predictive value. 

### 3.2. Prevalence of Reduced Serum DAO Activity in Patients with Symptoms of Histamine Intolerance

**Figure 3:**
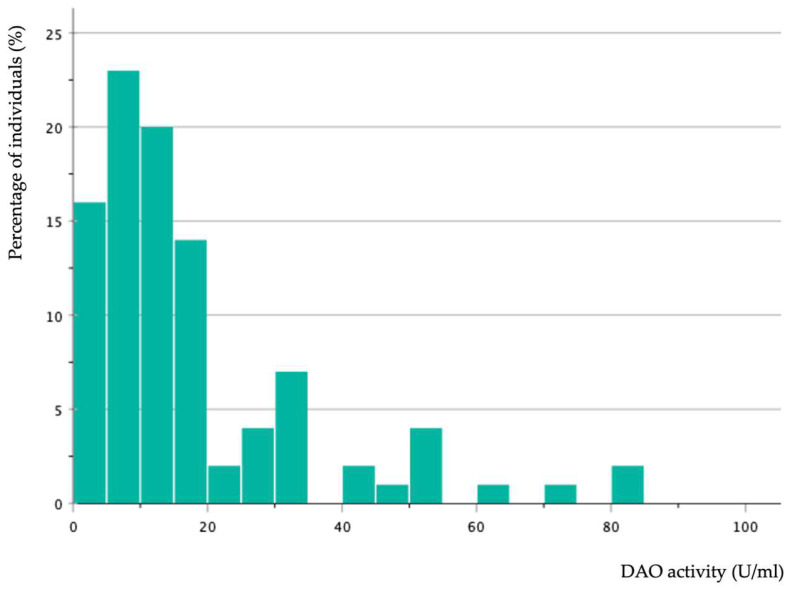
Frequency distribution of serum DAO activity values in patients with symptoms of histamine intolerance.

Figure 3 shows the frequency distribution of serum DAO activity levels in patients with symptoms of histamine intolerance. Only 43% of individuals had values below 10 U/mL, which is considered the cut-off value for DAO deficiency in the available enzymatic tests. In more detail, and according to the classification proposed in other studies [2,19,23,26,27], 36 patients had DAO activity values comprised between 3 and 10 (i.e., DAO deficiency) and 7 displayed levels below 3 (highly DAO-deficient). However, it is important to consider that DAO activity in the majority (75% of the total sample) was below 20 U/mL. These results indicate that the current cut-off limits could require revision, and further studies are necessary to establish the accurate distribution of serum DAO activity both in the healthy population and in individuals with histamine intolerance. In fact, the clinical validity of the measurement of serum DAO activity has been questioned by some authors [25]. On the other hand, considering that an alteration in serum DAO activity levels would be expected if there is a genetic origin, the high enzymatic activity displayed by individuals with symptoms of histamine intolerance included in this study could be attributed to other acquired etiological factors.

**Figure 4:**
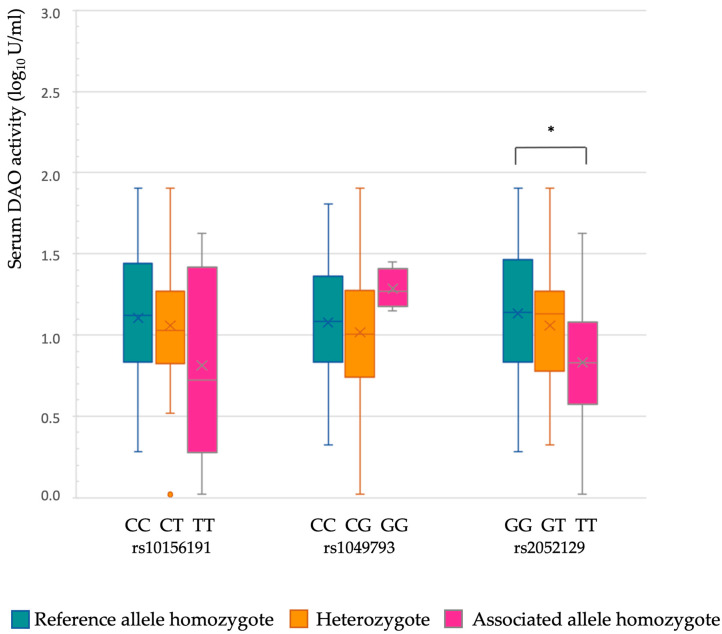
Distribution of logarithmic transformation of serum DAO activity data according to the allelic load of each different gene variant. * Asterisk indicates statistically significant differences (*p* < 0.05), according to the ANOVA and subsequent Tukey post-hoc tests. Variables of sex and age were not confounding factors according to a general linear model (*p* > 0.05).

Within the patients with symptoms of histamine intolerance harboring one or more of the three variants of the *AOC1* gene (*n* = 79), only 34 individuals showed DAO enzymatic deficiency (<10 U/mL), together with eight non-carriers. Overall, the serum DAO activity values were not directly associated with the number of gene variants carried per patient. Figure 4 shows the distribution of logarithmic transformation of serum DAO activity data according to the allelic dose of each variant. Age and sex were not confounders in any of the genetic variants assessed (*p* > 0.05). For rs2052129, the variant affecting the promoter region, it was observed that DAO activity was statistically lower in individuals with two DAO deficiency-associated alleles (TT homozygotes; *p* = 0.033). It should be noted that the prevalence of this genetic variant in TT homozygosis was statistically higher in patients than in the control group (Table 3). These results have also been corroborated when comparing the genotypes of patients stratified by their DAO activity levels (<3, 3–10, >10 U/mL). Thus, according to Table 5, the number of patients with the TT genotype in the rs2052129 variant is higher within the groups displaying DAO deficiency (*p* = 0.039). No differences were observed among the other genotypes within these cohorts for the rest of the genotypes.

These results agree with those of Maintz et al. (2011), who showed that patients carrying the T allele in variant rs2052129 had lower DAO activity than those carrying the G allele [15]. On the other hand, Ayuso et al. (2007) found that carriers of the variant rs1049793, whether heterozygous or homozygous, displayed a lower DAO activity than non-carriers [6]. However, García-Martin et al. (2022) did not find any type of influence of the genetic variants rs10156191, rs1049793 and rs1049742 on DAO activity [34]. Nevertheless, the relationship between DAO deficiency and the allelic load of the rs2052129 variant should be taken with caution because the validity of the test for serum DAO activity determination is not yet fully elucidated.

### 3.3. Correlation between the Number and Type of Symptoms of Histamine Intolerance and DAO-Encoding Gene Variants

**Figure 5:**
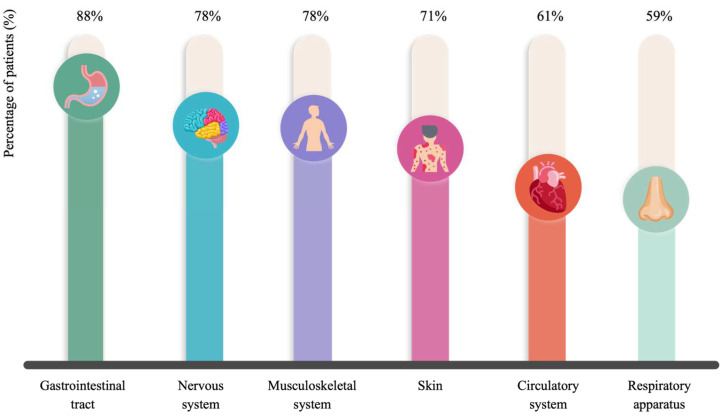
Percentage of patients affected by each category of symptoms associated with histamine intolerance.

The symptoms of histamine intolerance are numerous and multifaceted, reflecting the widespread distribution of the four histamine receptors within the human organism. Figure 5 shows the percentages of patients affected by each symptom category, with gastrointestinal, neurological and musculoskeletal disorders being the most frequently reported. Headache, bloating and fatigue were registered in more than 60% of patients, followed by pruritus, flushing, rhinitis, muscular pain, constipation and hypotension. The mean number of symptoms per patient was 8.0 ± 2.7, with 99% of individuals simultaneously suffering three or more complaints. Schnedl et al. (2019), who retrospectively evaluated the symptoms in a group of 133 histamine-intolerant patients, reported that 97% presented more than three symptoms, the most common being bloating, postprandial fullness, diarrhea, abdominal pain, dizziness and headache; the mean number of symptoms per patient was 11.1 ± 4.8 [4]. Similarly, the main symptoms of histamine intolerance found in the study of Sánchez-Pérez et al. (2022) were bloating and headache [9].

A literature review shows some studies that have reported a correlation between DAO-encoding gene SNVs and the occurrence of certain clinical manifestations. García-Martin et al. found that rs1049793 was associated with the severity of ulcerative colitis in a group of Caucasian adults [37], and rs10156191 and rs2052129 with the risk for migraine [16]. Meza-Velázquez et al. (2016) found that the rs1049793 variant was associated with rhinitis in a group of Mexican children and the occurrence of migraine in Mexican mothers [33,38]. Additionally, in a case-control study in a North Indian population, Kaur et al. (2020) demonstrated that variants rs2052129 and rs10156191 were statistically associated with migraine [14]. Recently, a prospective cohort study performed by Ponce Díaz-Reixa et al. (2023) has demonstrated a relation between the presence of minor alleles in rs2052129 and rs10156191 with a greater severity of obstructive urinary symptoms according to the mean scores of the International Prostate Symptom Score (IPSS) questionnaire (including symptoms such as the feeling of incomplete bladder emptying, frequency of urination, intermittency of urine stream, urgency of urination, weak stream, hesitation, and waking at night to urinate) [39]. Contrarily, the current study did not find any statistically significant relation between the number of genetic variants considered and the number of symptoms of histamine intolerance (*p* > 0.05). A relationship between any of the symptom categories and the frequency of any of the four genetic variants was not found alone or in combination. Similarly, Maintz et al. (2011) and García-Martín et al. (2022) did not observe any significant association of DAO SNVs with any particular symptom of histamine intolerance or with migraine, respectively, in patients with DAO deficiency [15,34]. In the present study, the presence of symptoms and their frequency were assessed, but their intensity was not considered. In future studies dealing with the relationship between genetics and symptoms, the intensity of the symptomatology measured by validated rating scales should also be considered.

**Figure 6:**
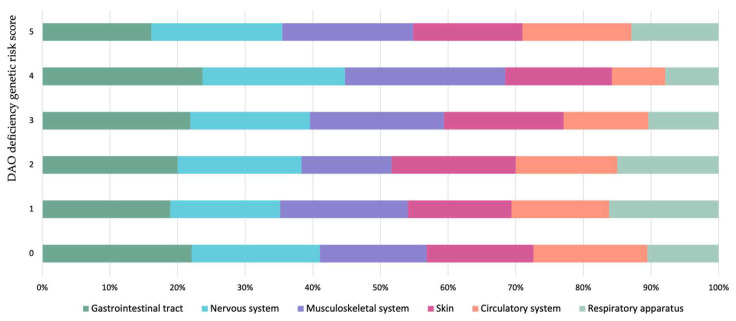
Distribution of symptoms according to the DAO deficiency genetic risk score (DAO Score), consisting of the sum of all risk alleles by giving a rating of 0 for the absence of any DAO deficiency-associated allele, 1 for the presence of one DAO deficiency-associated allele (heterozygosis) and 2 for the presence of two DAO deficiency-associated alleles (homozygosis) at each of the positions analyzed in the *AOC1* gene.

Moreover, in the current study, the clinical manifestations of the patients did not seem to depend on the allelic profile, as there were no significant associations between the number of symptoms and allele homozygosity or heterozygosity. In fact, as depicted in Figure 6, the distribution of each symptom category remains consistent across all genetic risk scores, regardless of the DAO Score load. 

## 4. Conclusions

The findings in this study suggest that identifying the number of allelic variants alone may not be sufficient as a suitable diagnostic biomarker for histamine intolerance caused by DAO deficiency. Regarding each of the SNVs, no significant differences were found in their appearance between the group of patients and healthy controls. In addition, the rs1049742 variant had a notably low incidence in both groups, thus rendering it an unlikely variant to be considered in diagnosing DAO deficiency.

However, when considering the status of the alleles associated with DAO deficiency, more homozygous alleles (carrying TT alleles) were observed in the patient than in the control group, especially in the cases of rs10156191 and rs2052129. The latter is located in the promoter region. Moreover, a slightly but statistically higher percentage of patients had a high genetic risk score (4–5), which could reflect the cumulative effect of carrying multiple DAO deficiency-associated SNVs and a high number of homozygous variants. Nevertheless, no correlation was found between the number of *AOC1* genes variants nor a specific variant in the *AOC1* gene and the type and number of symptoms associated with histamine intolerance or with serum DAO activity values. Although a significantly reduced DAO activity was observed in patients homozygous for rs2052129. 

One of the main drawbacks of the present study was the lack of data regarding serum DAO activity in control individuals and the divergence in the distribution of sexes in both study groups (although no differences were found when stratifying by sex). Moreover, it would have been of interest to measure the intensity of the clinical symptoms of the patients to assess any correlation with the presence of each genetic variant and/or the genetic risk score. The prevalence of genetic polymorphisms should also be studied based on the interindividual most intense symptomatology since it is possible that, even in the absence of genetic variants, symptoms of histamine intolerance appear when the origin of DAO deficiency is at the intestinal level. Additionally, a larger sample size, both for patients and controls, would have allowed a more accurate analysis and identification of haplotypes. These limitations should be brought to mind in further studies. Despite these limitations, this preliminary study provides cutting-edge data in the field of genetic background of histamine intolerance due to DAO deficiency. In particular, the results seem to indicate that carrying multiple DAO deficiency-associated gene variants, disregarding the rs1049742 variant, and a high load of risk alleles (homozygous) is more relevant than the mere presence of one or more SNVs. Therefore, in clinical practice, it becomes relevant that the mere presence of an altered variant would not serve as a diagnostic marker of histamine intolerance. Instead, emphasis should be placed on the genetic load, considering the use of a DAO deficiency genetic risk score as a potential predictive tool able to discriminate histamine-intolerant individuals due to a genetic etiology. However, this subject warrants more in-depth research to establish a predictive value for these DAO deficiency-related genetic variants.

## Figures and Tables

**Table 1:** Information of AOC1 gene (NM_001091.4) variants and the allelic and genotype frequencies in the European population.

dbSNV ID	Variant	Allelic Frequency ALFA *		Allelic Frecuency gnomAD ^¥^		Genotype Frequency		
rs10156191	c.47C > T (p.Thr16Met)	C	T	C	T	CC	CT	TT
		0.7393	0.2607	0.7392	0.2608	0.547	0.385	0.068
rs1049742	c.995C > T (p.Ser332Phe)	C	T	C	T	CC	CT	TT
		0.9275	0.0725	0.9219	0.0781	0.860	0.134	0.005
rs1049793	c.1990C > G (p.His664Asp)	C	G	C	G	CC	CG	GG
		0.6972	0.3029	0.7047	0.2953	0.486	0.422	0.092
rs2052129	c.-691G > T (promoter region)	G	T	G	T	GG	GT	TT
		0.7638	0.2362	0.7605	0.2395	0.583	0.361	0.056

* Allelic frequency alpha for the European Population according to Phan et al., 2020 [17]; ^¥^ Allelic frequency gnomAD v4.0.0 (GRCh38) for the European (non-Finnish) population.

**Table 2:** Symptoms associated with histamine intolerance included in the anamnesis questionnaire [3,5].

Organ/System	Symptoms
Circulatory system	Hypotonia, tachycardia, dizziness
Gastrointestinal tract	Bloating, abdominal pain, constipation, diarrhea, flatulence, colic, vomiting, reflux, postprandial fullness
Musculoskeletal system	Muscle pain, articular pain, cramps, fatigue
Nervous system	Migraine, headache, vertigo, attention deficit, lack of concentration, lack of memory
Respiratory apparatus	Rhinitis, rhinorrhea, nasal congestion, sneezing, asthma
Skin	Pruritus, flushing, eczemas, swelling

**Table 3:** DAO (*AOC1*) genotypes in patients with symptoms of histamine intolerance and in the healthy control group.

SNV	Genotype	Patients *n* = 100	Controls *n* = 100	*p*-Value *	*p*-Value **
rs10156191	C/C #	48	61	0.065	0.037
	C/T	40	35		
	T/T	12	4		
rs1049742	C/C #	82	87	0.329	0.316
	C/T	17	13		
	T/T	1	0		
rs1049793	C/C #	41	50	0.201	0.733
	C/G	55	45		
	G/G	4	5		
rs2052129	G/G #	49	53	0.572	<0.001
	G/T	32	47		
	T/T	19	0		

# The most common allelic frequency in the general population, according to Phan et al., 2020 [17]. * *p*-Value of the comparison of the number of individuals harboring *AOC1*-reference genotype versus the two DAO deficiency-associated genotypes (i.e., CC vs. CT + TT for rs10156191 and rs1049742, CC vs. CG + GG for rs1049793 and GG vs. GT + TT for rs2052129) between the patient and control groups. ** *p*-Value of the comparison of the number of individuals harboring *AOC1*-reference genotype plus heterozygous DAO deficiency-associated genotype versus homozygous DAO deficiency-associated genotype (i.e., CC + CT vs. TT for rs10156191 and rs1049742, CC + CG vs. GG for rs1049793 and GG + GT vs. TT for rs2052129) between the patient and control groups.

**Table 4:** Different combinations of genotypes detected in control and patient populations.

Thr16Met rs10156191	His664Asp rs1049793	Promoter Region rs2052129	Control Group (*n* = 100)	Population Frequency (95% Cl)	Patients Group (*n* = 100)	Population Frequency (95% Cl)	*p*- Value *
CC	CC	GG	28	28 (19.2–36.8)	21	21 (13.02–28.9)	0.324
CC	CC	GT	12	11 (4.87–17.1)	7	7 (2–12)	0.335
CC	CG	GG	16	16 (8.81–23.2)	18	18 (10.47–25.5)	0.851
CC	GG	GG	1	1 (0–2.95)	2	2 (0–4.7)	1
CC	CG	GT	3	3 (0–6.3)	0	0	0.324
CT	CC	GG	2	2 (0–4.7)	0	0	0.497
CT	CC	GT	7	7 (2–12)	5	5 (0.73–9.3)	0.767
CT	CG	GG	2	2 (0–4.7)	7	7 (2–12)	0.170
CT	CG	GT	22	22 (6.41–19.6)	17	17 (1.35–10.65)	0.476
CT	GG	GG	3	3 (0–6.3)	1	1 (0–2.95)	0.621
TT	CC	GT	1	1 (0–2.95)	1	1 (0–2.95)	1
TT	CG	GT	2	2 (0–4.7)	1	1 (0–2.95)	1
TT	GG	GG	1	1 (0–2.95)	0	0	1
CT	CC	TT	0	0	4	4 (0.16–7.8)	0.121
CT	CG	TT	0	0	5	5 (0.16–7.8)	0.059
CT	GG	GT	0	0	1	1 (0–2.95)	1
TT	CC	TT	0	0	3	3 (0–6.34)	0.246
TT	CG	TT	0	0	7	7 (0–2.95)	0.014

* *p*-Value of the comparison of the different combinations of genotypes between the patient and control groups according to Fisher’s Exact Test.

**Table 5:** DAO (*AOC1*) genotypes in patients with symptoms of histamine intolerance and its DAO serum activity levels.

SNV	Genotype	DAO Activity			*p*-Value *	*p*-Value **
		<3 U/mL	3–10 U/mL	>10 U/mL		
rs10156191	C/C #	3	14	31	0.214	0.504
	C/T	1	18	21		
	T/T	1	6	5		
rs1049793	C/C #	2	13	26	0.545	0.211
	C/G	3	25	27		
	G/G	0	0	4		
rs2052129	G/G #	2	17	30	0.694	0.039
	G/T	2	9	21		
	T/T	1	12	6		

# The most common allelic frequency in the general population, according to Phan et al., 2020 [17]. * *p*-Value of the comparison of the number of individuals harboring *AOC1*-reference genotype versus the two DAO deficiency-associated genotypes (i.e., CC vs. CT + TT for rs10156191 and rs1049742, CC vs. CG + GG for rs1049793 and GG vs. GT + TT for rs2052129) between patients according to their DAO activity levels. ** *p*-Value of the comparison of the number of individuals harboring *AOC1*-reference genotype plus heterozygous DAO deficiency-associated genotype versus homozygous DAO deficiency-associated genotype (i.e., CC + CT vs. TT for rs10156191 and rs1049742, CC + CG vs. GG for rs1049793 and GG + GT vs. TT for rs2052129) between patients according to their DAO activity levels.

## Data Availability

The original contributions presented in the study are included in the article, further inquiries can be directed to the corresponding authors.
